# Supplementary material for: Loss of Detection of sgN Precedes Viral Abridged Replication in COVID-19-Affected Patients—A Target for SARS-CoV-2 Propagation
Source: Int J Mol Sci. 2022 Feb 9;23(4):1941. doi: 10.3390/ijms23041941 (PMC8878976; doi:10.3390/ijms23041941)
Supplement: Supplementary file 1 [file ijms-23-01941-s001.zip › ijms-1557890-supplementary.pdf]

Supplemental Information

Loss of detection of sgN precedes viral abridged replication in COVID19-affected patients – a target for SARS-CoV-2 propagation

Veronica Ferrucci[1,2,14], Pasqualino de Antonellis[1,2,14], Fabrizio Quarantelli[2], Fatemeh Asadzadeh[1,2], Francesca Bibbò[1,2], Roberto Siciliano[1], Carmen Sorice[1,2], Ida Pisano[1], Barbara Izzo[1,2], Carmela Di Domenico[1], Angelo Boccia[1], Maria Vargas[3], Biancamaria Pierri[4], Maurizio Viscardi[4], Sergio Brandi[4], Giovanna Fusco[4], Pellegrino Cerino[4], Livia De Pietro[5], Ciro Furfaro [6], Leonardo Antonio Napolitano[6], Giovanni Paolella[1,2], Lidia Festa[7,8], Stefania Marzinotto[9], Maria Concetta Conte[7], Ivan Gentile[8], Giuseppe Servillo[3], Francesco Curcio[9], Tiziana De Cristofaro[10,11], Francesco Broccolo[12] , Ettore Capoluongo[1,2,13] and Massimo Zollo[1,2,13*]

1 CEINGE Biotecnologie Avanzate, Naples, 80145, Italy.
2 Dipartimento di Medicina Molecolare e Biotecnologie Mediche (DMMBM), 'Federico II' University of Naples, Naples, 80131, Italy.
3 Department of Neurosciences, Reproductive Sciences and Odontostomatology, University of Naples Federico II, Naples, Italy.
4 Istituto Zooprofilattico Sperimentale del Mezzogiorno, Naples, 80055, Italy.
5 U.O.C. Pneumologia, Ospedale Boscotrecase- Asl Napoli3 Sud, 80042 Boscotrecase-Naples, Italy.
6 Dipartimento di Medicina di Laboratorio e Anatomia Patologica, Asl Napoli 3 Sud 80035, Naples, Italy
7 Unità Speciale di Continuità Assistenziale (USCA), Asl Benevento, 82100, Italy.
8 Dipartimento di Medicina Clinica e Chirurgia, AOU Federico II, Naples, Italy
9 Dipartimento di Medicina di Laboratorio, Università degli Studi di Udine, 33100, Udine, Italy
10 IEOS-Institute of Experimental Endocrinology and Oncology G. Salvatore, National Research Council, Naples, 80131, Italy.
11 BioMol Laboratories srl, Naples, 80146, Italy.
12 Laboratory of Molecular Microbiology & Virology, School of Medicine, University of Milano-Bicocca, Monza, 20900, Italy.
13 DAI Medicina di Laboratorio e Trasfusionale, AOU Azienda Ospedaliera 'Federico II', Naples, 80131, Italy.
14 These authors contributed equally.
***Corresponding author**. Prof. Massimo Zollo. Email: massimo.zollo@unina.it

***Corresponding author**. Email: massimo.zollo@unina.it

Supplementary Figures

Supplementary Figure S1

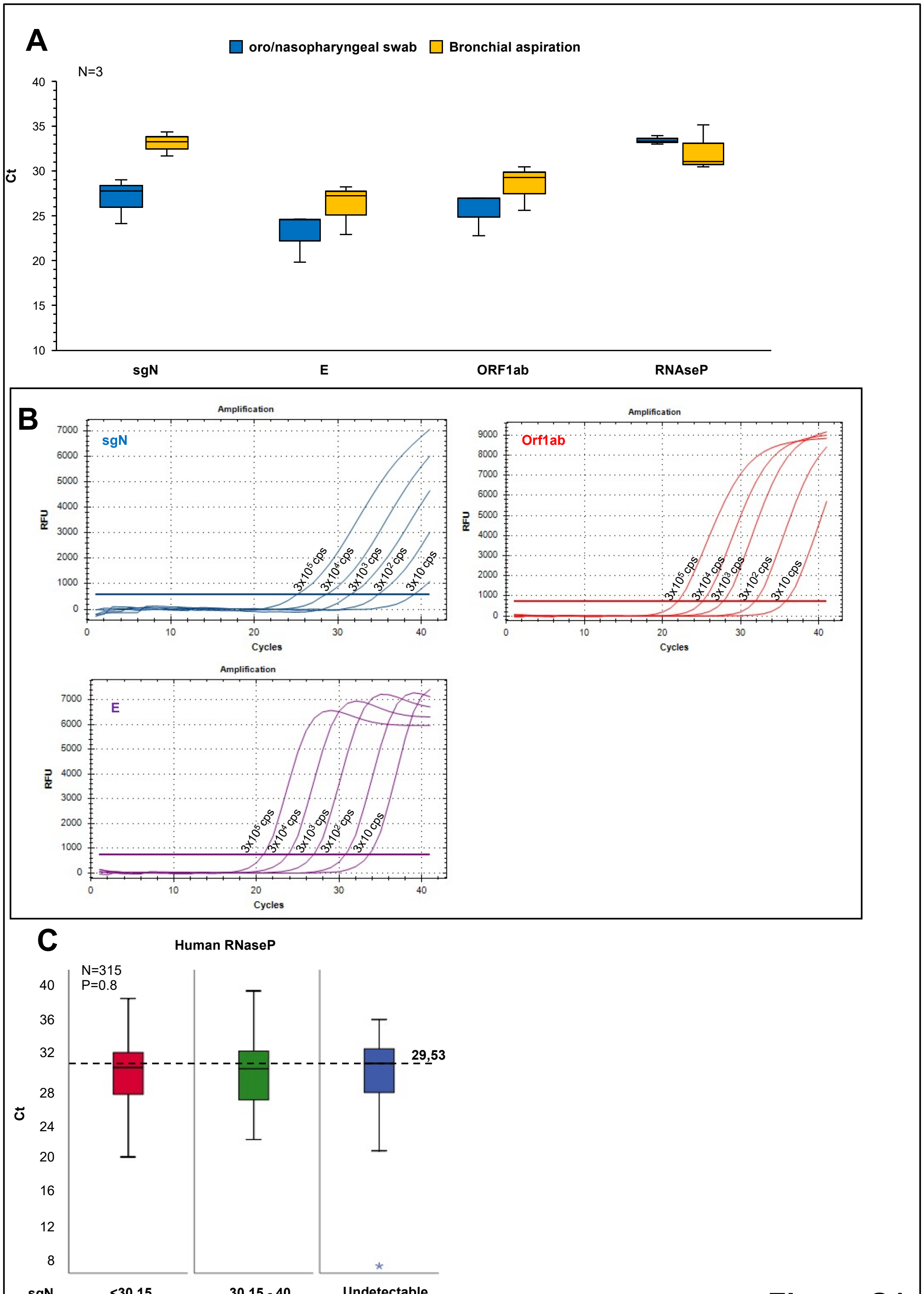

Figure S1

Supplementary Figure S1. SARS-CoV-2 Viral3 kit to detect sgN. Related to Figures 1. (A) Detection of viral sgN, gene E, gene ORF1ab and human RNAse P gene using the SARS-CoV-2 Viral3 kit for oro/nasopharyngeal swabs (orange) and bronchial aspirate samples (orange) in 3 hospitalized COVID19-positive patients. Mean Cq values are shown. **(B)** qPCR assays for the sensitivity of the SARS-CoV-2 Viral3 kit. Human 2019-nCoV strain 2019-nCoV/Italy INMI1 RNA was used as reference to test the sensitivity. Serial dilutions (from 3×10^5 to 3×10) of Human 2019-nCoV strain 2019-nCoV/Italy INMI1 RNA) were used. cps, standard copy particles. **(C)** Samples obtained from oro/nasopharyngeal swabs from COVID19-positive patients (N = 315) were stratified into three groups according to the median Cq values of sgN (sgN Cq median = 33.51). The first group consisted of those samples where Cq values for sgN were below the median value (i.e., Cq <30.51; 99 samples, in red). The second group of samples were characterized by Cq values of sgN ranged from the Cq median value (30.51) to 40 (96 samples, in green). Third group comprised samples in which sgN was not detectable (i.e., Cq >40; 120 samples, in blue). One-way analysis of variance (ANOVA) was used through IBM SPSS Statistics to determine the cut-off for sgN detection. SgN was detected in these samples independent of human RNAseP detection (P <0.8).

Supplementary Figure S2.

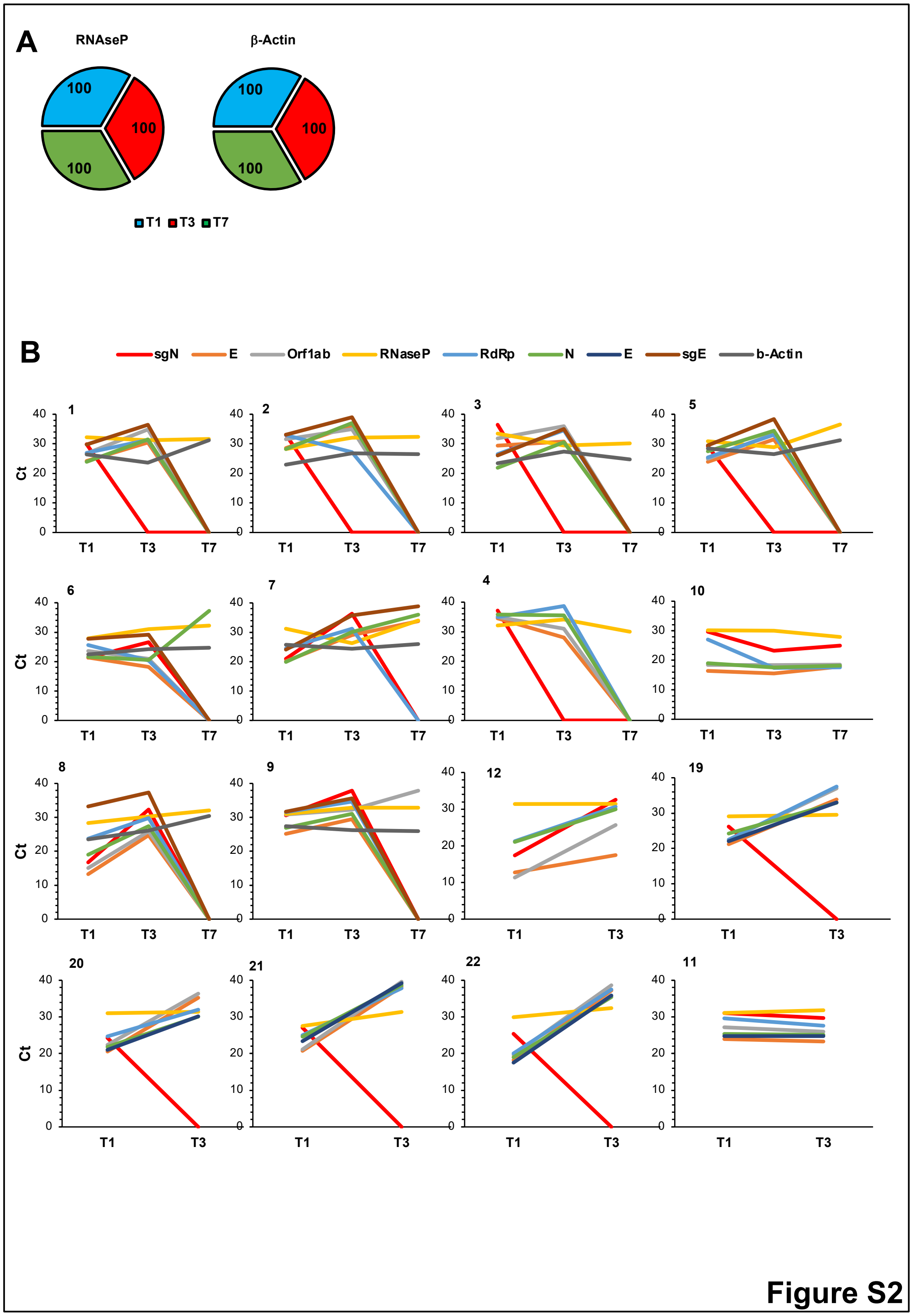

Supplementary Figure S2. Loss of detection of sgN precedes SARS-CoV-2 replication failure in home-isolated COVID19-affected patients. Related to Figures 2A-B. (A) Pie charts showing the proportions (%) of positivity of the oro/nasopharyngeal samples from home-isolated patients to human RNAse P and β-actin genes at the different times (blue, first swab [n = 16]; red, second swab collected after 3 days [n = 16]; green, third swab collected after 7 days [n = 10]). **(B)** Line graphs showing changes over time of the Cq values related to viral sgN (red), sgE (brown), gene N (green), gene E (orange), gene ORF1ab (light gray), RdRp (light blue), and human RNAse P (yellow) and β-actin (dark gray) genes in the oro/nasopharyngeal samples from 16 home-isolated patients.

Supplementary Figure S3.

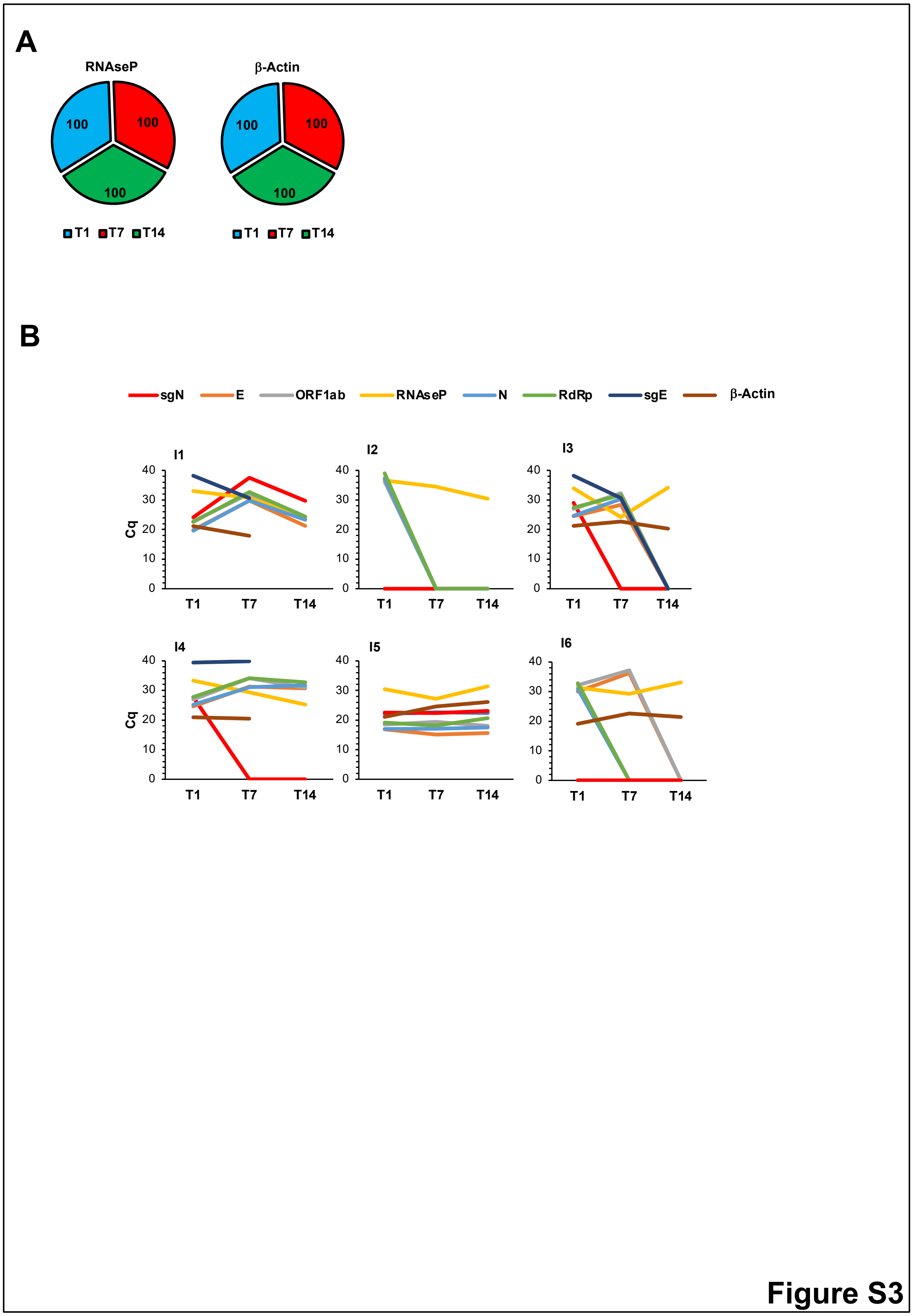

Supplementary Figure S3. Loss of detection of sgN precedes SARS-CoV-2 replication failure in home-isolated COVID19-affected patients. Related to Figures 2C-D. (A) Pie chart showing the proportions (%) of positivity of the oro/nasopharyngeal samples from 6 hospitalized COVID19-positive patients to human RNAse P gene at the different times (blue, first swab; red, second swab collected after 7 days; green, third swab collected after 14 days). **(B)** Line graphs showing the changes over time of the Cq values related to viral sgN (red), gene N (light blue), gene E (orange), gene ORF1ab (light gray), and RdRp (green), human RNAse P gene (yellow), sgE (dark blue and orange) and β-Actin (brown) in the oro/nasopharyngeal samples from 6 hospitalized patients.

Supplementary Figure S4.

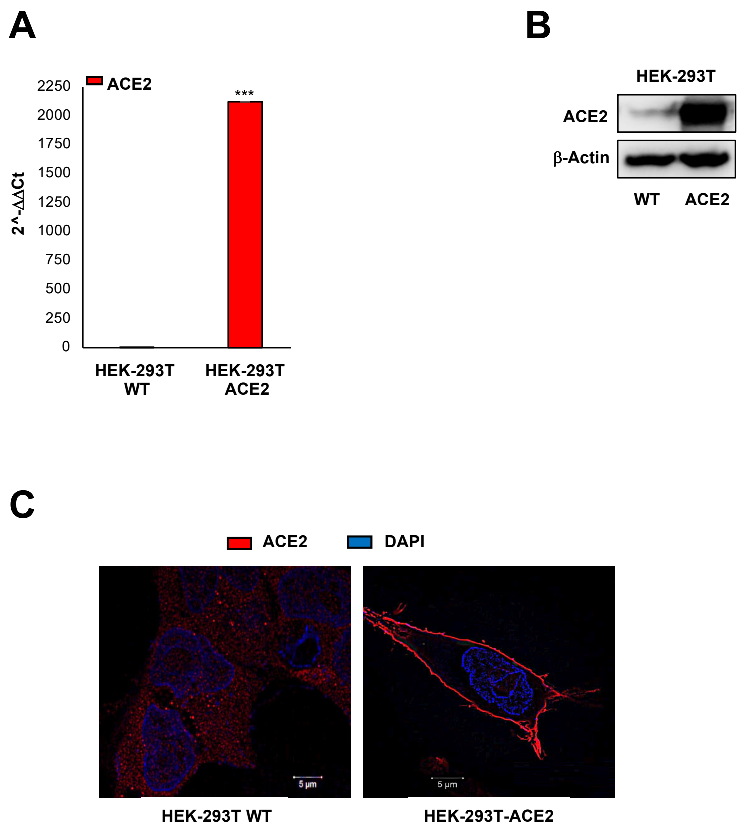

Figure S4

Supplementary Figure S4. HEK-293T overexpressing ACE2 cellular model. Related to Figures 3C-E, 4A-B. (A) Quantification of mRNA abundance relative to that of HEK-293T cells (2−ΔΔCt) for the ACE2 gene. RT-PCR analysis with SYBR Green of RNA extracted from HEK-293T wild-type (WT) and HEK-293T–overexpressing ACE2 cells. Data are means ±SD. ***P <0.001 (unpaired two-tailed Student's t test; n = 3 independent experiments per group). **(B)** Representative immunoblotting (using antibodies against the indicated proteins) of human HEK-293T cells and HEK-293T stable clones overexpressing ACE2. B-Actin was used as the loading control. (**C)** Immunofluorescence staining with an antibody against the human ACE2 (red) protein in human HEK-293T cells and HEK-293T stable clones overexpressing ACE2. DAPI was used for nuclei. The SIM image was acquired with Elyra 7 and processed with the Zeiss ZEN software (blue edition). Magnification, ×63. Scale bars: 5 μm.

Supplementary Figure S5.

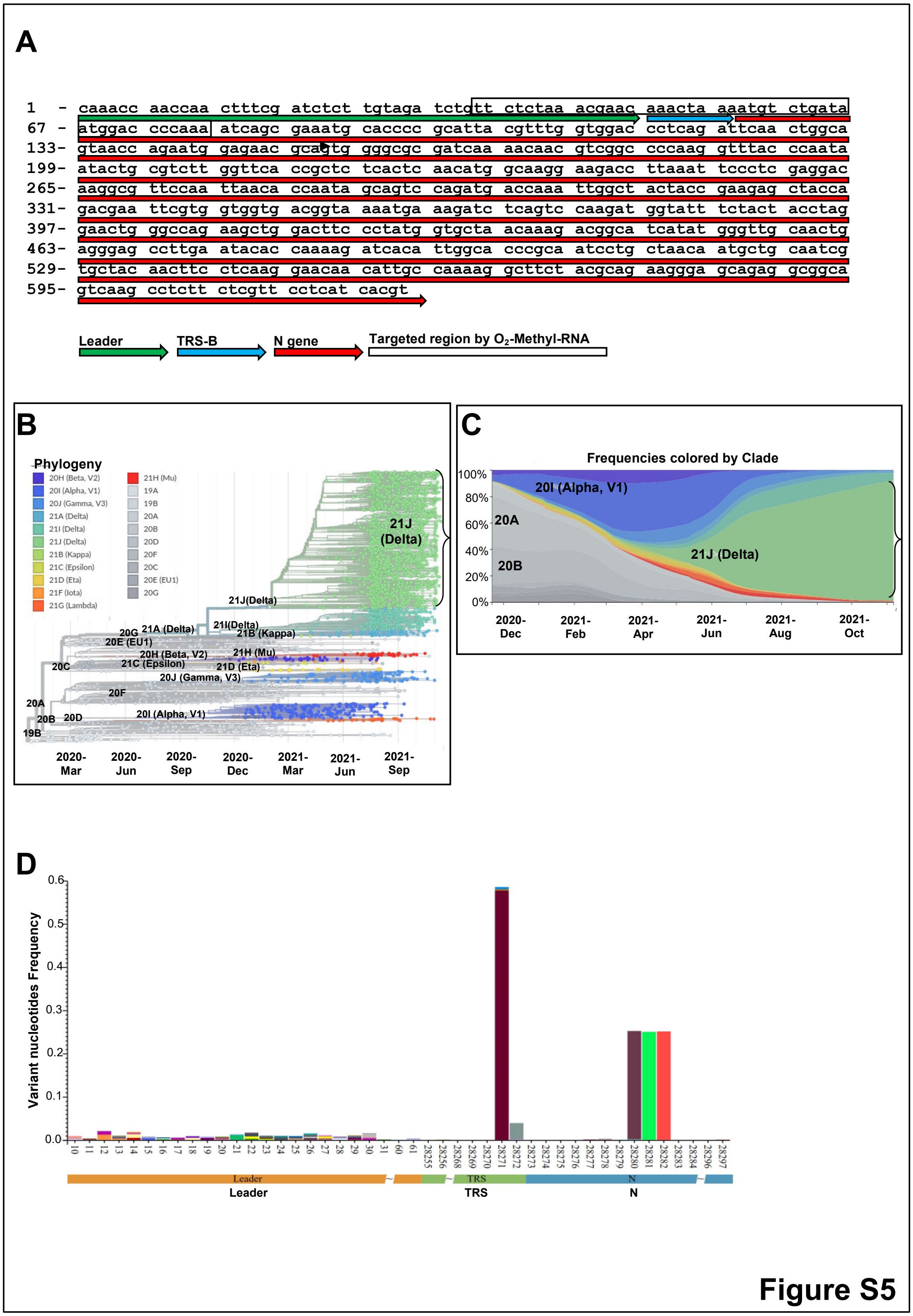

Figure S5

Supplementary Figure S5. 2'-*O*-methyl RNA against sgN sequence. Related to Figure 3A-B. (A) Sanger sequencing of the sgN region of SARS-CoV-2. Green arrow indicates the leader sequence, blue arrow indicates the TRS upstream gene N, and red arrow indicates gene N. The region recognized by 2'-*O*-methyl antisense RNA is highlighted in yellow. **(B, C)** Clade 21J is now the predominant form of Delta, with an estimated ~90% global frequency. **(D)** Frequencies of variant nucleotides calculated on a collection of about 350,000 sequences. Dataset downloaded from Global Initiative on Sharing Avian Influenza Data (GISAID) on November 15, 2021.

Supplementary Tables

Supplementary Table S1: Related to Figure 1. Cq values obtained for 50 oro/nasopharyngeal swabs from COVID19-positive patients according to the SARS-CoV-2 Viral3 kit and the Allplex 2019-nCoV assay.

**Code**	**Cq value**						
	**SARS-CoV-2 Viral3 kit**				**Allplex 2019-nCoV assay**		
	**E gene**	**sgN transcript**	**ORF1ab gene**	**RNAse P gene**	**E gene**	**N gene**	**RdRP/S gene**
S8	16.36	20.37	15.83	33.28	13.84	15.58	14.16
S48	17.78	25.60	16.79	30.22	15.51	16.66	15.62
S4	15.15	18.56	14.54	30.56	12.08	13.38	15.65
S82	20.66	28.96	19.76	36.21	17.06	18.20	16.05
S37	19.38	24.17	18.49	30.75	13.30	15.67	16.64
S94	19.04	30.32	19.50	30.84	13.50	14.83	16.69
S16	16.31	22.26	16.20	31.43	13.79	15.73	16.74
S162	19.17	35.75	19.19	33.42	17.55	18.53	17.07
S33	18.88	23.82	17.82	30.80	17.34	18.56	17.18
S36	16.28	24.12	15.33	35.34	13.69	16.79	17.29
S35	17.26	24.02	16.62	31.80	16.60	16.48	17.33
S46	17.84	25.59	17.80	34.92	16.84	18.22	17.42
S43	19.37	24.63	18.52	31.58	16.88	18.78	17.46
S188	20.87	38.78	24.66	29.88	15.99	15.95	17.63
S6	16.54	19.28	16.00	31.48	16.58	17.68	17.67
S5	18.67	18.82	17.66	32.43	17.03	18.00	17.97
S11	15.80	20.82	16.31	31.15	15.78	15.92	18.34
S32	20.59	23.60	20.54	30.31	18.59	19.18	18.43
S23	18.47	22.73	17.53	32.01	16.74	16.91	18.53
S10	18.45	20.66	19.65	24.36	17.95	18.14	18.57
S27	20.58	23.31	19.50	31.98	19.04	19.77	18.75
S3	16.73	17.83	16.03	31.71	17.20	17.73	18.82
S25	18.71	23.09	19.06	34.34	18.68	19.87	18.88
S7	18.05	19.90	17.58	34.08	17.03	17.27	18.94
S62	21.45	26.65	23.51	38.09	19.23	20.55	19.09
S113	22.67	31.48	22.24	33.66	19.30	20.53	19.16
S59	22.20	26.50	21.13	34.97	19.56	21.16	19.28
S28	18.31	23.41	17.27	29.76	17.58	18.50	19.30
S29	18.75	23.56	19.09	34.11	17.90	17.60	19.68
S69	18.38	27.49	18.18	29.52	17.82	18.52	19.71
S42	19.15	24.57	19.19	32.09	19.07	20.46	20.07
S34	19.01	23.86	19.17	32.48	18.68	18.33	20.19
S100	22.07	30.67	21.77	33.91	20.54	21.73	20.37
S64	20.18	26.96	19.73	34.76	19.08	19.38	20.67
S9	18.78	20.63	18.21	31.34	18.63	18.67	20.70
S60	21.18	26.51	19.89	35.12	20.82	19.64	20.71
S70	21.18	27.65	21.16	38.10	18.84	19.21	20.94
S12	19.46	20.96	19.64	31.86	18.83	19.03	21.21

S61	19.59	26.54	19.72	33.18	19.43	19.54	21.25
S67	21.28	27.30	21.02	34.16	19.69	19.86	21.39
S73	26.40	27.75	26.50	35.30	24.31	24.95	23.14
S84	24.14	29.04	24.19	32.04	23.95	24.12	25.81
S120	28.05	31.87	28.04	36.70	28.22	28.68	26.32
S106	25.72	31.05	25.38	32.90	25.08	26.34	26.45
S163	30.14	35.75	29.64	36.23	28.30	27.76	26.56
S121	31.98	31.90	30.87	38.98	28.59	27.68	26.91
S156	29.34	35.06	29.15	35.59	28.79	28.89	27.15
S119	27.28	31.84	27.44	34.63	25.27	25.79	27.16
S96	28.64	30.49	28.91	27.29	26.11	25.69	28.21
S161	30.29	35.39	30.30	33.30	27.03	27.30	28.22

Supplementary Table S2. Related to Figure 1. Cq values obtained for 12 oro/nasopharyngeal swabs from negative COVID19-tested people according to the SARS-CoV-2 Viral3 kit and the Allplex 2019-nCoV assay.

**Code**	**Cq values**						
	**SARS-CoV-2 Viral3 kit**				**Allplex 2019-nCoV assay**		
	**E gene**	**sgN transcript**	**ORF1ab gene**	**RNAse P gene**	**E gene**	**N gene**	**RdRP/S gene**
N1	N.A.	N.A.	N.A.	38.26	N.A.	N.A.	N.A.
N2	N.A.	N.A.	N.A.	34.59	N.A.	N.A.	N.A.
N3	N.A.	N.A.	N.A.	34.82	N.A.	N.A.	N.A.
N4	N.A.	N.A.	N.A.	31.12	N.A.	N.A.	N.A.
N6	N.A.	N.A.	N.A.	37.75	N.A.	N.A.	N.A.
N7	N.A.	N.A.	N.A.	25.58	N.A.	N.A.	N.A.
N8	N.A.	N.A.	N.A.	28.54	N.A.	N.A.	N.A.
N9	N.A.	N.A.	N.A.	23.17	N.A.	N.A.	N.A.
N10	N.A.	N.A.	N.A.	26.42	N.A.	N.A.	N.A.
N11	N.A.	N.A.	N.A.	30.46	N.A.	N.A.	N.A.
N12	N.A.	N.A.	N.A.	26.49	N.A.	N.A.	N.A.

N.A., not amplified (i.e., Cq value >40).

Supplementary Table S3. Related to Figure 1A-B. Cq values obtained from the 315 oro/nasopharyngeal swabs from COVID19-positive patients with the SARS-CoV-2 Viral3 kit.

Code	Cq value				Code	Cq value			
	E	sgN	ORF1ab	RNAse P		E	sgN	ORF1ab	RNAse P
**S1**	16.18	15.12	16.41	28.45	**S159**	26.61	35.31	26.44	27.75
**S2**	20.43	17.02	21.45	29.23	**S160**	26.61	35.31	26.44	27.75
**S3**	16.73	17.83	16.03	31.71	**S161**	30.29	35.39	30.30	33.30
**S4**	15.15	18.56	14.54	30.56	**S162**	19.17	35.75	19.19	33.42
**S5**	18.67	18.82	17.66	32.43	**S163**	30.14	35.75	29.64	36.23
**S6**	16.54	19.28	16.00	31.48	**S164**	28.31	36.57	29.10	26.02
**S7**	18.05	19.90	17.58	34.08	**S165**	28.31	36.57	29.10	26.02
**S8**	16.36	20.37	15.83	33.28	**S166**	29.16	36.59	28.94	28.64
**S9**	18.78	20.63	18.21	31.34	**S167**	30.27	36.62	30.73	29.67
**S10**	18.45	20.66	19.65	24.36	**S168**	14.74	36.77	14.09	22.18
**S11**	15.80	20.82	16.31	31.15	**S169**	14.74	36.77	14.09	22.18
**S12**	19.46	20.96	19.64	31.86	**S170**	25.85	36.94	25.22	28.00
**S13**	15.12	21.79	17.46	26.59	**S171**	29.05	36.98	29.18	26.40
**S14**	15.12	21.79	17.46	26.59	**S172**	26.98	37.00	26.67	32.00
**S15**	15.16	22.05	14.65	27.00	**S173**	26.66	37.19	25.42	23.01
**S16**	16.31	22.26	16.20	31.43	**S174**	26.66	37.19	25.42	23.01
**S17**	17.70	22.34	18.73	26.82	**S175**	29.90	37.24	30.46	25.64
**S18**	17.70	22.34	18.73	26.82	**S176**	29.90	37.24	30.46	25.64
**S19**	17.35	22.41	17.52	24.75	**S177**	27.62	37.42	27.43	32.00
**S20**	17.35	22.41	17.52	24.75	**S178**	30.77	37.56	31.18	31.61
**S21**	20.04	22.61	20.32	26.64	**S179**	30.77	37.56	31.18	31.61
**S22**	20.04	22.61	20.32	26.64	**S180**	27.13	37.60	27.27	32.08
**S23**	18.47	22.73	17.53	32.01	**S181**	33.59	37.72	34.24	30.88
**S24**	23.13	23.07	23.73	28.47	**S182**	30.40	37.80	31.35	31.67
**S25**	18.71	23.09	19.06	34.34	**S183**	25.01	37.89	25.46	29.65
**S26**	17.70	21.43	17.94	29.25	**S184**	25.01	37.89	25.46	29.65
**S27**	20.58	23.31	19.50	31.98	**S185**	28.16	37.98	28.84	31.13
**S28**	18.31	23.41	17.27	29.76	**S186**	28.16	37.98	28.84	31.13
**S29**	18.75	23.56	19.09	34.11	**S187**	22.38	38.25	23.04	26.27
**S30**	18.87	23.59	19.13	26.55	**S188**	20.87	38.78	24.66	29.88
**S31**	18.87	23.59	19.13	26.55	**S189**	28.56	38.85	28.01	28.93
**S32**	20.59	23.60	20.54	30.31	**S190**	26.06	38.88	25.22	30.53
**S33**	18.88	23.82	17.82	30.80	**S191**	30.17	38.90	29.02	32.24
**S34**	19.01	23.86	19.17	32.48	**S192**	32.64	38.99	32.48	34.09
**S35**	17.26	24.02	16.62	31.80	**S193**	30.14	39.02	30.21	33.30
**S36**	16.28	24.12	15.33	35.34	**S194**	30.34	39.12	30.15	32.11
**S37**	19.38	24.17	18.49	30.75	**S195**	27.54	39.19	26.83	33.10
**S38**	20.05	24.22	20.97	30.60	**S196**	24.32	N.A.	23.17	25.21
**S39**	20.05	24.22	20.97	30.60	**S197**	26.50	N.A.	25.73	31.21
**S40**	21.42	24.40	22.02	26.14	**S198**	26.52	N.A.	26.52	26.43
**S41**	21.42	24.40	22.02	26.14	**S199**	26.63	N.A.	25.84	28.65
**S42**	19.15	24.57	19.19	32.09	**S200**	26.88	N.A.	27.61	33.86
**S43**	19.37	24.63	18.52	31.58	**S201**	26.94	N.A.	26.39	30.14
**S44**	20.51	25.03	20.63	28.18	**S202**	27.02	N.A.	25.57	25.00

**S45**	20.52	25.43	21.19	30.08
**S46**	17.84	25.59	17.80	34.92
**S47**	19.26	23.94	19.15	30.60
**S48**	17.78	25.60	16.79	30.22
**S49**	20.33	25.71	21.10	29.35
**S50**	19.37	25.78	19.73	25.70
**S51**	17.01	25.88	17.19	22.79
**S52**	17.01	25.88	17.19	22.79
**S53**	25.46	25.98	25.57	30.39
**S54**	19.61	26.00	20.50	30.43
**S55**	20.33	26.11	21.56	28.38
**S56**	20.15	26.15	20.81	28.35
**S57**	17.47	26.39	17.99	27.70
**S58**	17.47	26.39	17.99	27.70
**S59**	22.20	26.50	21.13	34.97
**S60**	21.18	26.51	19.89	35.12
**S61**	19.59	26.54	19.72	33.18
**S62**	21.45	26.65	23.51	38.09
**S63**	20.04	26.70	20.05	31.00
**S64**	20.18	26.96	19.73	34.76
**S65**	21.38	27.24	22.25	26.96
**S66**	21.38	27.24	22.25	26.96
**S67**	21.28	27.30	21.02	34.16
**S68**	22.07	27.40	21.94	31.11
**S69**	18.38	27.49	18.18	29.52
**S70**	21.18	27.65	21.16	38.10
**S71**	21.60	27.70	21.03	30.09
**S72**	18.59	27.74	25.70	27.47
**S73**	26.40	27.75	26.50	35.30
**S74**	20.46	27.88	20.10	31.23
**S75**	20.67	28.11	22.61	29.13
**S76**	20.15	28.13	20.94	28.82
**S77**	20.15	28.13	20.94	28.82
**S78**	19.28	28.20	19.32	27.46
**S79**	24.13	28.64	24.52	29.42
**S80**	18.59	28.75	17.64	25.18
**S81**	18.59	28.75	17.64	25.18
**S82**	20.66	28.96	19.76	36.21
**S83**	16.36	29.02	16.64	19.82
**S84**	24.14	29.04	24.19	32.04
**S85**	24.92	29.23	24.69	31.00
**S86**	18.45	29.29	26.88	28.45
**S87**	18.75	29.52	19.69	20.76
**S88**	24.25	29.53	24.07	31.00
**S89**	23.79	29.66	23.34	31.90
**S90**	24.32	29.82	24.83	27.84
**S91**	18.52	30.18	17.72	25.35
**S92**	18.52	30.18	17.72	25.35
**S93**	22.44	30.24	22.75	30.25
**S94**	19.04	30.32	19.50	30.84
**S203**	27.06	N.A.	26.25	31.53
**S204**	27.10	N.A.	26.11	27.56
**S205**	27.15	N.A.	26.35	30.88
**S206**	28.01	N.A.	28.08	21.13
**S207**	28.12	N.A.	26.85	22.56
**S208**	28.15	N.A.	29.20	31.10
**S209**	28.54	N.A.	27.03	23.46
**S210**	29.44	N.A.	28.43	21.68
**S211**	29.47	N.A.	29.67	32.39
**S212**	29.48	N.A.	29.80	32.75
**S213**	29.66	N.A.	29.36	31.44
**S214**	30.18	N.A.	30.60	28.63
**S215**	30.35	N.A.	31.17	34.03
**S216**	30.73	N.A.	29.51	25.68
**S217**	30.77	N.A.	31.03	32.09
**S218**	30.86	N.A.	29.52	31.64
**S219**	31.10	N.A.	32.20	29.02
**S220**	31.22	N.A.	31.75	32.61
**S221**	31.29	N.A.	31.42	31.98
**S222**	31.39	N.A.	32.22	24.57
**S223**	31.39	N.A.	31.47	32.37
**S224**	31.45	N.A.	31.36	32.47
**S225**	31.47	N.A.	31.92	31.60
**S226**	31.51	N.A.	32.14	33.78
**S227**	31.63	N.A.	31.82	34.04
**S228**	31.69	N.A.	31.58	32.29
**S229**	31.71	N.A.	31.85	30.55
**S230**	31.95	N.A.	30.63	23.50
**S231**	32.00	N.A.	32.02	26.08
**S232**	32.15	N.A.	31.31	23.07
**S233**	32.16	N.A.	33.77	26.18
**S234**	32.20	N.A.	32.54	35.03
**S235**	32.22	N.A.	32.35	30.50
**S236**	32.40	N.A.	33.78	34.06
**S237**	32.40	N.A.	33.23	33.86
**S238**	32.53	N.A.	32.19	27.44
**S239**	32.69	N.A.	32.55	23.08
**S240**	32.75	N.A.	32.02	31.48
**S241**	32.75	N.A.	32.76	33.57
**S242**	32.89	N.A.	33.02	27.95
**S243**	32.98	N.A.	33.18	31.03
**S244**	33.05	N.A.	31.81	27.02
**S245**	33.10	N.A.	32.11	31.61
**S246**	33.12	N.A.	33.02	28.33
**S247**	33.13	N.A.	33.20	31.20
**S248**	33.15	N.A.	33.43	30.83
**S249**	33.15	N.A.	32.97	32.37
**S250**	33.23	N.A.	32.75	32.23
**S251**	33.25	N.A.	32.22	26.29
**S252**	33.30	N.A.	33.04	33.50

**S95**	16.89	30.42	18.91	19.71	**S253**	33.34	N.A.	34.05	29.96
**S96**	28.64	30.49	28.91	27.29	**S254**	33.38	N.A.	33.56	30.59
**S97**	24.37	30.49	24.43	32.00	**S255**	33.44	N.A.	33.68	25.23
**S98**	23.24	30.49	22.81	32.08	**S256**	33.49	N.A.	33.07	27.39
**S99**	22.06	30.51	30.21	29.12	**S257**	33.67	N.A.	33.03	34.98
**S100**	22.07	30.67	21.77	33.91	**S258**	33.70	N.A.	32.72	21.89
**S101**	25.61	30.69	25.58	33.29	**S259**	33.74	N.A.	33.96	31.10
**S102**	21.08	30.76	20.53	29.81	**S260**	33.84	N.A.	34.09	26.58
**S103**	23.30	30.76	23.85	30.39	**S261**	33.85	N.A.	32.48	29.74
**S104**	21.36	30.84	22.62	22.84	**S262**	33.95	N.A.	33.95	26.49
**S105**	22.56	31.05	23.08	31.70	**S263**	34.09	N.A.	33.69	34.35
**S106**	25.72	31.05	25.38	32.90	**S264**	34.10	N.A.	33.74	28.07
**S107**	19.69	31.06	29.08	28.42	**S265**	34.10	N.A.	33.35	30.66
**S108**	28.16	31.07	28.53	32.00	**S266**	34.14	N.A.	35.26	34.66
**S109**	22.14	31.11	27.65	29.21	**S267**	34.19	N.A.	35.11	28.51
**S110**	23.80	31.27	23.59	30.10	**S268**	34.23	N.A.	34.87	30.28
**S111**	18.95	31.29	19.45	23.45	**S269**	34.25	N.A.	34.61	22.51
**S112**	26.14	31.42	25.98	28.36	**S270**	34.25	N.A.	32.92	31.49
**S113**	22.67	31.48	22.24	33.66	**S271**	34.25	N.A.	35.98	28.15
**S114**	21.48	31.58	22.66	25.70	**S272**	34.26	N.A.	35.01	30.14
**S115**	22.10	31.65	21.05	21.73	**S273**	34.33	N.A.	33.62	26.48
**S116**	22.10	31.65	21.05	21.73	**S274**	34.36	N.A.	35.03	32.93
**S117**	24.28	31.68	25.58	31.35	**S275**	34.37	N.A.	34.51	20.43
**S118**	25.25	31.70	25.24	32.01	**S276**	34.48	N.A.	35.23	32.24
**S119**	27.28	31.84	27.44	34.63	**S277**	34.50	N.A.	34.41	31.16
**S120**	28.05	31.87	28.04	36.70	**S278**	34.50	N.A.	34.60	31.16
**S121**	31.98	31.90	30.87	38.98	**S279**	34.59	N.A.	34.40	33.33
**S122**	25.74	31.98	26.20	30.02	**S280**	34.68	N.A.	33.89	30.21
**S123**	24.21	32.09	23.83	30.06	**S281**	34.73	N.A.	34.49	34.39
**S124**	23.43	32.25	23.94	28.28	**S282**	34.73	N.A.	35.13	33.30
**S125**	26.33	32.25	26.27	31.44	**S283**	34.90	N.A.	35.58	31.01
**S126**	20.19	32.36	20.10	27.42	**S284**	35.06	N.A.	34.38	27.45
**S127**	25.41	32.40	24.54	29.38	**S285**	35.12	N.A.	33.81	21.71
**S128**	21.69	32.46	24.69	23.94	**S286**	35.12	N.A.	37.19	30.61
**S129**	26.89	32.53	26.45	29.14	**S287**	35.16	N.A.	34.67	7.06
**S130**	20.78	32.58	19.59	25.15	**S288**	35.18	N.A.	35.74	25.19
**S131**	20.78	32.58	19.59	25.15	**S289**	35.20	N.A.	36.04	30.15
**S132**	24.49	32.68	26.02	28.34	**S290**	35.34	N.A.	36.49	31.28
**S133**	26.83	32.83	31.92	33.50	**S291**	35.40	N.A.	35.57	26.87
**S134**	18.68	32.85	18.97	26.55	**S292**	35.47	N.A.	35.91	35.66
**S135**	29.20	32.97	29.47	38.03	**S293**	35.76	N.A.	36.34	32.81
**S136**	27.45	33.04	29.30	29.65	**S294**	35.83	N.A.	37.33	28.48
**S137**	27.57	33.06	27.97	30.07	**S295**	35.94	N.A.	38.10	30.48
**S138**	25.67	33.32	26.17	31.17	**S296**	36.00	N.A.	36.58	32.74
**S139**	25.67	33.32	26.17	31.17	**S297**	36.08	N.A.	34.14	32.90
**S140**	23.63	33.35	23.65	23.13	**S298**	36.13	N.A.	37.50	29.16
**S141**	23.63	33.35	23.65	23.13	**S299**	36.16	N.A.	36.35	31.19
**S142**	24.67	33.44	31.25	33.90	**S300**	36.18	N.A.	36.31	30.95
**S143**	24.86	33.45	23.99	30.00	**S301**	36.21	N.A.	36.70	31.10
**S144**	27.30	33.53	27.19	28.84	**S302**	36.28	N.A.	35.74	34.68

**S145**	20.64	33.71	21.34	24.45	**S303**	36.39	N.A.	37.25	26.97
**S146**	23.56	33.72	23.53	30.08	**S304**	36.71	N.A.	38.21	26.99
**S147**	20.34	33.77	19.20	23.64	**S305**	36.74	N.A.	38.25	35.29
**S148**	20.34	33.77	19.20	23.64	**S306**	36.98	N.A.	36.69	28.51
**S149**	24.95	33.92	25.60	33.28	**S307**	37.13	N.A.	36.05	31.11
**S150**	27.78	33.96	28.00	31.88	**S308**	37.19	N.A.	38.09	31.35
**S151**	27.72	34.07	27.81	30.14	**S309**	37.61	N.A.	36.69	28.06
**S152**	28.87	34.18	29.79	33.89	**S310**	38.07	N.A.	36.32	30.28
**S153**	22.87	34.24	21.34	21.81	**S311**	38.30	N.A.	40.20	25.99
**S154**	22.87	34.24	21.34	21.81	**S312**	39.79	N.A.	38.81	35.17
**S155**	22.07	34.58	21.88	31.90	**S313**	39.79	N.A.	37.27	29.62
**S156**	29.34	35.06	29.15	35.59	**S314**	40.23	N.A.	40.40	30.22
**S157**	19.16	35.14	19.35	26.84	**S315**	40.26	N.A.	40.08	30.52
**S158**	19.16	35.14	19.35	26.84					

N.A., not amplified (i.e., Cq >40)

Supplementary Table S4. Related to Figure 1D,F,H. Cq values for sgE and β-actin obtained from the single cohort of 122 oro/nasopharyngeal swabs from COVID19-positive patients analyzed using Taqman qPCR.

Code	Cq value		Code	Cq value		Code	Cq value		Code	Cq value	
	sgE	β-actin		sgE	β-actin		sgE	β-actin		sgE	β-actin
**S44**	27.37	26.50	**S312**	39.40	26.95	**S261**	N.A.	24.48	**S274**	N.A.	25.44
**S45**	27.50	27.75	**S191**	39.49	26.34	**S239**	N.A.	26.2	**S267**	N.A.	27.54
**S49**	26.92	25.39	**S200**	39.65	28.35	**S250**	N.A.	30.57	**S276**	N.A.	28.45
**S54**	26.19	27.27	**S199**	39.74	29.76	**S241**	N.A.	30.78	**S266**	N.A.	33.50
**S55**	27.36	25.92	**S219**	39.88	28.03	**S270**	N.A.	28.09	**S291**	N.A.	23.98
**S56**	26.80	25.39	**S243**	38.04	27.35	**S249**	N.A.	29.90	**S283**	N.A.	28.03
**S71**	30.75	28.07	**S256**	38.25	32.94	**S242**	N.A.	29.51	**S302**	N.A.	27.18
**S74**	40.4	30.28	**S212**	38.57	29.13	**S246**	N.A.	27.11	**S288**	N.A.	25.24
**S98**	31.27	28.53	**S215**	38.81	29.84	**S257**	N.A.	24.47	**S292**	N.A.	33.38
**S105**	37.98	28.06	**S213**	39.17	29.19	**S252**	N.A.	26.87	**S271**	N.A.	25.56
**S112**	35.15	27.05	**S280**	39.45	27.76	**S247**	N.A.	29.69	**S289**	N.A.	26.91
**S127**	36.95	30.51	**S227**	38.73	30.58	**S237**	N.A.	30.55	**S307**	N.A.	28.49
**S137**	37.45	27.42	**S234**	38.34	31.15	**S265**	N.A.	29.04	**S300**	N.A.	27.74
**S144**	40.10	28.67	**S203**	N.A.	28.14	**S248**	N.A.	30.38	**S310**	N.A.	24.03
**S149**	33.92	27.80	**S201**	N.A.	29.33	**S254**	N.A.	27.94	**S293**	N.A.	30.41
**S150**	37.49	28.96	**S198**	N.A.	27.84	**S273**	N.A.	25.37	**S299**	N.A.	27.48
**S151**	34.30	26.84	**S218**	N.A.	27.33	**S263**	N.A.	31.51	**S290**	N.A.	22.25
**S166**	38.85	26.63	**S217**	N.A.	30.21	**S264**	N.A.	27.97	**S306**	N.A.	30.13
**S167**	40.10	26.15	**S224**	N.A.	30.52	**S262**	N.A.	24.45	**S301**	N.A.	28.23
**S180**	41.10	30.08	**S221**	N.A.	27.62	**S259**	N.A.	25.81	**S286**	N.A.	28.38
**S190**	38.03	26.79	**S223**	N.A.	29.47	**S253**	N.A.	26.29	**S303**	N.A.	27.48
**S238**	28.94	25.97	**S228**	N.A.	29.21	**S260**	N.A.	25.81	**S294**	N.A.	22.79
**S206**	31.03	28.27	**S220**	N.A.	27.22	**S297**	N.A.	30.51	**S298**	N.A.	27.30
**S275**	34.37	26.10	**S244**	N.A.	29.69	**S284**	N.A.	29.72	**S308**	N.A.	28.20
**S282**	36.72	28.70	**S229**	N.A.	25.35	**S279**	N.A.	27.47	**S295**	N.A.	27.27
**S208**	37.82	27.04	**S225**	N.A.	30.02	**S277**	N.A.	30.19	**S304**	N.A.	25.69
**S205**	38.18	28.11	**S231**	N.A.	30.60	**S281**	N.A.	33.87	**S305**	N.A.	30.99
**S211**	38.47	27.09	**S240**	N.A.	29.44	**S278**	N.A.	27.52	**S315**	N.A.	29.07
**S197**	38.87	28.86	**S245**	N.A.	28.46	**S287**	N.A.	25.94	**S311**	N.A.	31.67
**S236**	39.40	30.65	**S226**	N.A.	30.72	**S268**	N.A.	26.54			
**S235**	N.A.	29.60	**S272**	N.A.	29.32	**S314**	N.A.	25.23			

N.A., not amplified (i.e., Cq >40)

Supplementary Table S5. Related to Figure 2A-B. Cq values obtained from a cohort of 16 oro/nasopharyngeal swabs from home-isolated COVID19-infected patients analyzed according to scheduled times (i.e., 3-day intervals from the first swab) with the SARS-CoV-2 Viral3 kits (sgN, E gene, ORF1ab gene) and the Allplex 2019-nCoV assay (E, N, RdRp/S genes) and by Taqman qPCR (sgE, β-actin genes).

**Marker**	**Day**	**Cq value**															
		**#1**	**#2**	**#3**	**#4**	**#5**	**#6**	**#7**	**#8**	**#9**	**#10**	**#11**	**#12**	**#19**	**#20**	**#21**	**#22**
**SARS-CoV-2 Viral3 kit**																	
sgN transcript	1	29.87	33.08	36.46	37.16	29.21	21.38	20.92	16.7	30.58	29.67	31.07	17.38	26.22	24.26	27.07	25.41
	3	N.A.	N.A.	N.A.	N.A.	N.A.	26.59	36.35	32.29	37.87	23.25	29.71	32.58	N.A.	N.A.	N.A.	N.A.
	7	N.A.	N.A.	N.A.	N.A.	N.A.	N.A.	N.A.	N.A.	N.A.	24.95	-	-	-	-	-	-
E gene	1	24.22	28.52	29.34	34.5	23.89	21.38	20.15	13.3	25.13	16.44	23.96	12.75	21.23	20.57	20.77	18.25
	3	30.43	36.28	30.76	28.04	31.49	18.16	29.03	24.76	29.5	15.54	23.32	17.48	33.87	35.23	38.28	37.23
	7	N.A.	N.A.	N.A.	N.A.	N.A.	N.A.	33.78	N.A.	N.A.	17.83	-	-	-	-	-	-
Orf1ab gene	1	26.77	31.55	31.88	35.04	24.84	23.62	23.18	15.05	30.85	18.47	27.18	11.34	22.51	22.39	21.15	19.68
	3	34.84	34.94	35.95	30.98	33.37	20.98	35.83	25.87	32.32	18.37	25.99	25.67	37.03	36.38	39.65	38.64
	7	N.A.	N.A.	N.A.	N.A.	N.A.	N.A.	N.A.	N.A.	37.91	18.52	-	-	-	-	-	-
RNAse P gene	1	32.23	28.27	33.49	32.14	30.91	27.89	31.21	28.34	31	30.13	31.11	31.42	29.11	31.04	27.53	29.93
	3	31.2	32.07	29.46	34.11	28.87	31.07	26.31	30.24	32.87	30.02	31.86	31.49	29.56	31.39	31.37	32.42
	7	31.7	32.4	30.23	30.01	36.56	32.25	34	32.1	32.87	27.87	-	-	-	-	-	-
**Allplex 2019-nCoV assay**																	
RdRp/S gene	1	26.93	32.94	26.55	34.94	25.45	25.64	24.46	23.78	31.55	27.03	29.61	21.26	22.47	24.71	25.03	20.05
	3	31.34	27.15	34.6	38.74	33.12	20.38	31.23	29.84	34.64	17.41	27.64	30.67	37.51	31.98	37.86	37.49
	7	N.A.	N.A.	N.A.	N.A.	N.A.	N.A.	N.A.	N.A.	N.A.	17.66	-	-	-	-	-	-
N gene	1	23.96	28.35	21.91	35.81	27.44	21.60	19.90	18.94	26.92	19.03	25.38	21.09	24.25	21.85	24.6	19.02
	3	31.5	37.01	30.55	35.56	34.42	20.59	30.17	27.42	31.05	17.6	25.14	29.98	32.97	30.09	38.71	35.45
	7	N.A.	N.A.	N.A.	N.A.	N.A.	37.23	36	N.A.	N.A.	18.21	-	-	-	-	-	-
E gene	1	24.55	29.38	21.74	34.37	24.98	22.30	21.19	19.04	26.62	20.71	24.79	20.14	22.06	21.01	23.37	17.54
	3	32.29	38.74	31.03	35.52	31.26	20.54	31.23	25.6	31.39	17.3	24.78	32.35	33.06	30.18	39.22	35.86
	7	N.A.	N.A.	N.A.	N.A.	N.A.	N.A.	36.05	N.A.	N.A.	17.94	-	-	-	-	-	-
**Taqman qPCR**																	
sgE transcript	1	29.84	33.08	26.11	-	29.55	27.78	24.11	33.17	31.63	-	-	-	-	-	-	-
	3	36.42	38.97	35.01	-	38.4	29.18	35.68	37.35	35.64	-	-	-	-	-	-	-
	7	N.A.	N.A.	N.A.	-	N.A.	N.A.	38.81	N.A.	N.A.	-	-	-	-	-	-	-
β-actin gene	1	26.42	22.96	23.42	-	28.45	22.42	25.68	23.52	27.42	-	-	-	-	-	-	-
	3	23.63	26.83	27.39	-	26.53	24.21	24.39	26.11	26.18	-	-	-	-	-	-	-
	7	31.2	26.58	24.72	-	31.23	24.73	25.99	30.44	25.97	-	-	-	-	-	-	-

N.A., not amplified (i.e., Cq >40)

Supplementary Table S6. Related to Figure 2C-D. Cq values obtained from a cohort of 6 oro/nasopharyngeal swabs from hospitalized COVID19-positive patients analyzed according to scheduled times (i.e., 7-day intervals from the first swab) with the SARS-CoV-2 Viral3 kits (sgN, E gene, ORF1ab gene) and the Allplex 2019-nCoV assay (E, N, RdRp/S genes) and by Taqman qPCR (sgE, β-actin genes).

Marker	Days	Cq value					
		I1	I2	I3	I4	I5	I6
**SARS-CoV-2 Viral3 kits**							
sgN transcript	1	24.13	N.A.	29.01	27.76	22.06	N.A.
	7	37.47	N.A.	N.A.	N.A.	22.62	N.A.
	14	29.63	N.A.	N.A.	N.A.	22.44	N.A.
E gene	1	19.85	37.36	24.56	24.66	16.86	30.04
	7	29.76	N.A.	28.32	31.32	15.09	36.27
	14	21.24	N.A.	N.A.	30.67	15.64	N.A.
ORF1ab gene	1	22.79	36.2	26.95	26.98	18.47	32.16
	7	32.11	N.A.	32.31	34.18	19.37	37.2
	14	23.49	N.A.	N.A.	31.03	18.01	N.A.
RNAseP	1	33.01	36.6	33.93	33.31	30.44	31.34
	7	30.63	34.5	24.21	29.39	27.2	29.24
	14	24.05	30.37	34.12	25.22	31.42	33.14
**Allplex 2019-nCoV assay**							
E gene	1	20.55	38.46	25.44	25.85	17.26	30.44
	7	30.27	N.A.	29.34	32.1	16.26	37.76
	14	21.96	N.A.	N.A.	30.79	18	N.A.
N gene	1	19.57	37.1	24.61	25.21	17.01	30.92
	7	29.8	N.A.	30.31	31.17	17.11	N.A.
	14	23.25	N.A.	N.A.	31.77	17.52	N.A.
RdRp/S gene	1	22.54	39	27.43	27.8	19.12	32.88
	7	32.69	N.A.	31.64	34.16	18.13	N.A.
	14	24.32	N.A.	N.A.	32.71	20.67	N.A.
**Taqman qPCR**							
sgE transcript	1	38.15	-	39.13	39.45	22.58	39.55
	7	30.59	-	39.88	39.86	22.34	N.A.
	14	-	-	N.A.	-	23.09	N.A.
β-actin gene	1	21.15	-	21.22	20.93	21.07	19.16
	7	17.87	-	22.68	20.49	24.61	22.64
	14	-	-	20.28	-	26.08	21.41

N.A., not amplified (i.e., Cq values >40)
